# Transitions in Tobacco Product Use by U.S. Adults between 2013–2014 and 2014–2015: Findings from the PATH Study Wave 1 and Wave 2

**DOI:** 10.3390/ijerph15112515

**Published:** 2018-11-09

**Authors:** Karin A. Kasza, Nicolette Borek, Kevin P. Conway, Maciej L. Goniewicz, Cassandra A. Stanton, Eva Sharma, Geoffrey T. Fong, David B. Abrams, Blair Coleman, Liane M. Schneller, Elizabeth Y. Lambert, Jennifer L. Pearson, Maansi Bansal-Travers, Iilun Murphy, Yu-Ching Cheng, Elisabeth A. Donaldson, Shari P. Feirman, Shannon Gravely, Tara Elton-Marshall, Dennis R. Trinidad, Daniel A. Gundersen, Raymond S. Niaura, K. Michael Cummings, Wilson M. Compton, Andrew J. Hyland

**Affiliations:** 1 Department of Health Behavior, Roswell Park Comprehensive Cancer Center, Buffalo, NY 14263, USA; 2 Office of Science, Center for Tobacco Products, U.S. Food and Drug Administration, Silver Spring, MD 20993, USA; 3 National Institute on Drug Abuse, National Institutes of Health, Bethesda, MD 20892, USA; 4 Westat, Rockville, MD 20850, USA; 5 School of Public Health and Health Systems, University of Waterloo, Waterloo, ON N2L 3G1, Canada; 6 Department of Psychology, University of Waterloo, Waterloo, ON N2L 3G1, Canada; 7 Ontario Institute for Cancer Research, Toronto, ON M5G 0A3, Canada; 8 The Schroeder Institute for Tobacco Research and Policy Studies, Truth Initiative, Washington, DC 20001, USA; 9 Institute for Mental Health Policy Research, Centre for Addiction and Mental Health, London, ON M5T 1R8, Canada; 10 Dalla Lana School of Public Health, University of Toronto, Toronto, ON M5S 1A1, Canada; 11 Department of Epidemiology and Biostatistics, Schulich School of Medicine and Dentistry, Western University, London, ON N6A 3K7, Canada; 12 Ontario Tobacco Research Unit, Toronto, ON M5S 2S1, Canada; 13 Department of Family Medicine and Public Health, University of California, La Jolla, CA 92093, USA; 14 Department of Family Medicine and Community Health, Robert Wood Johnson Medical School, Rutgers, Somerset, NJ 08873, USA; 15 Survey and Data Management Core, Dana-Farber Cancer Institute, Boston, MA 02215, USA; 16 Department of Psychiatry & and Behavioral Sciences, Medical University of South Carolina, Charleston, SC 29425, USA

**Keywords:** tobacco, transition, population, longitudinal, epidemiology, cigarettes, cigars, hookah, smokeless tobacco, electronic nicotine delivery systems (ENDS)

## Abstract

In 2013–2014, nearly 28% of adults in the United States (U.S.) were current tobacco users with cigarettes the most common product used and with nearly 40% of tobacco users using two or more tobacco products. We describe overall change in prevalence of tobacco product use and within-person transitions in tobacco product use in the U.S. between 2013–2014 and 2014–2015 for young adults (18–24 years) and older adults (25+ years). Data from Wave 1 (W1, 2013–2014) and Wave 2 (W2, 2014–2015) of the Population Assessment of Tobacco and Health (PATH) Study were analyzed (*N* = 34,235). Tobacco product types were categorized into: (1) combustible (cigarettes, cigars, pipe tobacco, hookah), (2) noncombustible (smokeless tobacco, snus pouches, dissolvable tobacco), and (3) electronic nicotine delivery systems (ENDS). Transitions for individual combustible-product types, and for single- and multiple-product use, were also considered. Overall prevalence of current tobacco use decreased from 27.6% to 26.3%. Among W1 non-tobacco users, 88.7% of young adults and 95.8% of older adults were non-tobacco users at W2. Among W1 tobacco users, 71.7% of young adults transitioned, with 20.7% discontinuing use completely, and 45.9% of older adults transitioned, with 12.5% discontinuing use completely. Continuing with/transitioning toward combustible product(s), particularly cigarettes, was more common than continuing with/transitioning toward ENDS. Tobacco use behaviors were less stable among young adults than older adults, likely reflecting greater product experimentation among young adults. Relative stability of cigarette use compared to other tobacco products (except older adult noncombustible use) demonstrates high abuse liability for cigarettes.

## 1. Introduction

Cigarette smoking remains the leading cause of preventable death in the world and in the United States (U.S.), where it is responsible for more than 480,000 deaths each year [1] and annually costs the nation more than USD 300 billion in medical costs [2] and lost productivity due to premature death [1,3]. While the prevalence of cigarette smoking has declined in the U.S. [1] and most other high income countries (e.g., Australia, Canada, England, Sweden, and New Zealand) during the past several decades [4], the tobacco product marketplace has expanded in many countries [5], including in the U.S. where the prevalence of use of non-cigarette tobacco products, such as electronic nicotine delivery systems (ENDS), cigars, hookah (e.g., waterpipe, shisha), and smokeless tobacco (e.g., snus pouches, chewing tobacco), has been increasing [6,7,8,9,10,11]. Recent national data show that nearly 28% of U.S. adults (aged 18+ years) were current users of at least one of the aforementioned tobacco products in 2013–2014, with 18% of adults smoking cigarettes, and nearly 40% of tobacco users using more than one type of tobacco product [12]. 

Understanding how tobacco product use is changing over time, including the prevalence of never users, formers users, and users of combustible tobacco products, noncombustible tobacco products, and ENDS, along with within-person changes in tobacco product use, including adding use, stopping use, and switching among types of tobacco products used, is important in predicting downstream health risks of tobacco use in the population at large. While tobacco control policies and regulations vary among countries, an understanding of tobacco product transition behaviors occurring in the U.S. within its current regulatory environment can also be informative to other countries, particularly those with similarly expanding tobacco product markets [5]. For example, the extent to which tobacco users and nonusers transition in ENDS use over a one-year period in a largely unregulated ENDS market can be informative to a number of countries. In this paper, we report change in the overall prevalence of tobacco product use and within-person transitions in tobacco product use over a one-year period of time in the U.S. using a nationally representative sample of young adults (18–24 years of age) and older adults (25 years of age and older). 

## 2. Materials and Methods

### 2.1. Study Population

The Population Assessment of Tobacco and Health (PATH) Study is a large, nationally representative longitudinal study of tobacco use and health among adults and youths in the U.S. [13]. We report national cross-sectional estimates from 34,235 adults (18+ years of age) who participated in Wave 1 (W1, data collection conducted 12 September 2013 through 14 December 2014) or Wave 2 (W2, 23 October 2014 through 30 October 2015) of the PATH Study. Youths aged 17 years at W1, who were aged 18 years at W2 (N = 1915), were only included in the W2 cross-sectional estimates. We also report longitudinal estimates from 26,439 adults (18+ years of age at W1) who participated in both W1 and W2.

The PATH Study recruitment employed a stratified address-based, area-probability sampling design at W1 that oversampled adult tobacco users, young adults (18 to 24 years), and black adults. An in-person screener was used at W1 to select youths and adults from households for participation. At W1, the weighted response rate for the household screener was 54.0%. Among households that were screened, the overall weighted response rate for the Adult interview was 74.0% at W1 and 83.2% at W2. Data were collected using audio-computer assisted self-interviews administered in English or Spanish.

The PATH Study weighted estimates presented here are robust and representative of the non-institutionalized, civilian U.S. adult population through use of the probability sample and application of population and replicate weights that adjust for the complex study design characteristics (e.g., oversampling at W1) and nonresponse at W1 and W2. Further details regarding the PATH Study W1 design and methods are published elsewhere [13]. Details on survey interview procedures, questionnaires, sampling, weighting, and information on accessing the data are available online [14]. The PATH Study was conducted by Westat and approved by the Westat institutional review board (Institutional Review Board (IRB) Organization number IORG0000410, IRB number 00000695). All participants aged 18 and older provided informed consent.

### 2.2. Measures

#### 2.2.1. Types of Tobacco Products

At each wave, participants were asked about their use of 10 types of tobacco products that were grouped for analysis into three categories of products: (1) combustible (i.e., cigarettes, traditional cigars, cigarillos, filtered cigars, hookah, and pipe tobacco), (2) noncombustible (i.e., loose snus, moist snuff, dip, spit, chewing tobacco, snus pouches, dissolvable tobacco), and (3) ENDS. We looked at transitions among these categories, as well as among individual combustible tobacco product categories (i.e., cigarettes, any cigar, hookah, and pipe tobacco), noncombustible, and ENDS. Participants were provided with brief descriptions and pictures of each type of tobacco product except cigarettes.

#### 2.2.2. Use of Tobacco Products

For each type of tobacco product, participants were asked if they had ever used the product (even one or two puffs/times), how much they had used in their lifetime, and whether they now use the product every day, some days, or not at all (non-daily hookah users were additionally asked about their frequency of use). At each wave, current use for cigarettes was defined as having smoked at least 100 cigarettes in one’s lifetime and now smoking every day or somedays; for hookah as now smoking every day, somedays, usually weekly, or usually monthly (no threshold for units used); and for all other tobacco products as now smoking/using every day or somedays (no threshold for units used).

#### 2.2.3. Tobacco Product User Groups

Any current tobacco, no current tobacco, never tobacco, single-product, dual-product, and multiple-product user groups were defined as indicated in Table 1. Complete data were required when defining non-use for each tobacco product user group.

### 2.3. Statistical Analysis

Analyses were conducted with the use of Stata 14 software [15]. Estimates were weighted to represent the U.S. adult population and variances were estimated using the balanced repeated replication (BRR) method [16] with Fay’s adjustment set to 0.3 to increase estimate stability [17]. The logit-transformation method was used to calculate confidence intervals.

Cross-sectional prevalence estimates of the tobacco user groups shown in Table 1 were determined at W1 and at W2, by age group (i.e., among young adults aged 18–24 years at each wave (*N* = 9109 at W1; *N* = 8174 at W2), and among older adults aged 25+ years at each wave (*N* = 23,194 at W1; *N* = 20,183 at W2)). Chi-squared tests were used to determine whether prevalence for each user group differed between waves.

Within-person rates of transitions among tobacco user groups between W1 and W2, and classification in the same tobacco user group in both waves, were determined by age group (i.e., among young adults aged 18–24 years at W1 who were aged approximately 19–25 years at W2 (*N* = 7324), and among older adults aged 25+ years at W1 who were aged approximately 26+ years at W2 (*N* = 19,115)).

## 3. Results

### 3.1. Prevalence of Tobacco User Groups at Wave 1 and Wave 2

Population-level prevalences of tobacco user groups at W1 and W2, where the denominator for each group is the full population at each wave, are shown in Table 1, by age group, and differences in prevalences between waves are indicated. Prevalence of any current tobacco use decreased from 27.6% in W1 to 26.3% in W2 among all adults (*p* < 0.05, not shown in table), from 37.6% in W1 to 35.4% in W2 among young adults (*p* < 0.05), and from 26.0% to 25.0% among older adults (*p* < 0.05).

When considering the three broad tobacco user groups, the group with the greatest decrease in prevalence between waves was combustible only for both young adults (from 24.4% to 18.5%) and older adults (from 17.5% to 16.8%), and the group with the greatest increase in prevalence between waves was combustible + ENDS for young adults (from 6.2% to 9.1%) and ENDS only for older adults (from 0.9% to 1.3%). When considering the six tobacco product categories, prevalence of any single-product use and any multiple-product use each decreased between waves among young adults (*p* < 0.05), while among older adults, prevalence of any single-product use increased and any multiple-product use decreased (*p* < 0.05).

### 3.2. Within-Person Transitions in Tobacco Product Use Status between Wave 1 and Wave 2

**Figure 1:**
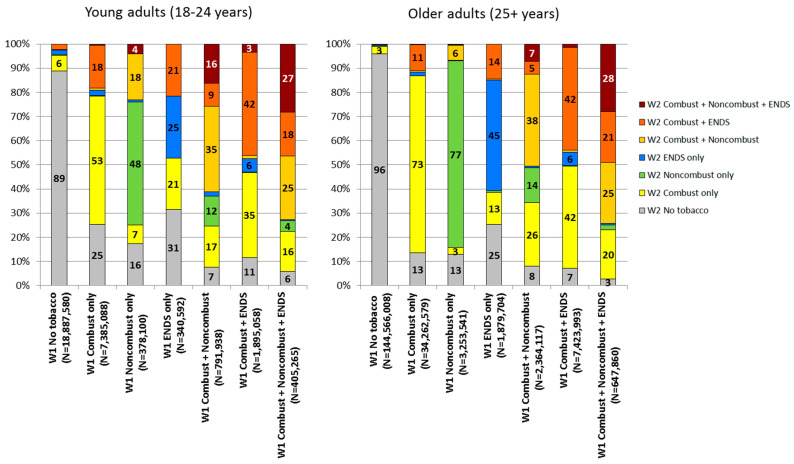
Transitions in current tobacco product use among young adults (aged 18-24 years) and older adults (aged 25+ years) in the United States: 2013–2014 to 2014–2015. Notes. Each bar in the figure represents a mutually exclusive tobacco product user group at Wave 1 (W1). N underneath each bar indicates the weighted population size for each tobacco product user group at W1 (i.e., the scale of the y-axis differs across bars). For each tobacco product user group at W1, transitions between W1 and Wave 2 (W2) are indicated by the colored distribution of each bar; for example, among the 18,887,580 young adult No tobacco users at W1, 89% continued No tobacco use at W2 and 6% transitioned to Combustible only use at W2. Current tobacco use defined as: for cigarettes, currently using everyday/somedays and smoked at least 100 cigarettes in lifetime; for hookah, currently using everyday/somedays/usually weekly/usually monthly; for all other products, currently using everyday/somedays. ‘Combustible’ includes cigarettes, traditional cigars, cigarillos, filtered cigars, hookah, pipe tobacco; ‘noncombustible’ includes smokeless tobacco, snus pouches, dissolvable tobacco; ‘ENDS’ includes e-cigarettes at each wave and e-products at W2. ‘No tobacco’ defined as no current use of any tobacco product. Estimates are suppressed when unweighted denominator <50 or RSE>30%; all estimates for ‘Noncombustible + ENDS’ are suppressed. Non-suppressed estimates <3 are not shown in the figure (for readability) but are shown in the appendix.

Within-person transitions among user groups based on the three broad tobacco product categories as defined in Table 1 are presented for young adults and older adults in Figure 1, with additional details provided for young adults in Supplemental Table S1 and additional details provided for older adults in Supplemental Table S2. The population-level overall impact of each transition reported below is a function of both the size of the tobacco user group at W1 and the size of the transition between W1 and W2. Thus, one must consider the relative size of the W1 user groups (shown in parentheses beside each subheading below) when considering the total contribution that each transition makes to overall change in population-level prevalences as reported above in Table 1.

#### 3.2.1. No Tobacco Users at Wave 1 (Accounting for 62.4% of the Young Adult Population (Table S1) and 74.0% of the Older Adult Population (Table S2))

Among young adults, nearly 90% of no tobacco users at W1 were still no tobacco users at W2, 6.3% transitioned to combustible only (with 1.7% transitioning to cigarettes only, 1.4% transitioning to cigars only, and 2.2% transitioning to hookah only, Table S3), 0.5% transitioned to noncombustible only, 1.8% transitioned to ENDS only, and 2.1% transitioned to combustible + ENDS (Figure 1 and Table S1).

Among older adults, more than 95% of no tobacco users at W1 were still no tobacco users at W2, 3.1% transitioned to combustible only (with 1.8% transitioning to cigarettes only, 0.8% transitioning to cigars only, and 0.2% transitioning to hookah only, Table S4), 0.2% transitioned to noncombustible only, 0.4% transitioned to ENDS only, and 0.2% transitioned to combustible + ENDS (Figure 1 and Table S2).

#### 3.2.2. Combustible Only Users at Wave 1 (Accounting for 24.4% of the Young Adult Population (Table S1) and 17.5% of the Older Adult Population (Table S2))

Among young adults, more than 50% of combustible only users at W1 were still combustible only users at W2, 17.6% transitioned to combustible + ENDS, and 25.1% transitioned to no tobacco use (Figure 1 and Table S1). Among older adults, more than 70% of combustible only users at W1 were still combustible only users at W2, 10.6% transitioned to combustible + ENDS, and 13.4% transitioned to no tobacco use (Figure 1 and Table S2).

#### 3.2.3. Noncombustible Only Users at Wave 1 (Accounting for 1.2% of the Young Adult Population (Table S1) and 1.6% of the Older Adult Population (Table S2))

Among young adults, almost 50% of noncombustible only users at W1 were still noncombustible only users at W2, 17.9% transitioned to combustible + noncombustible, and 16.4% transitioned to no tobacco use (Figure 1 and Table S1). Among older adults, more than 75% of noncombustible only users at W1 were still noncombustible only users at W2, 2.8% transitioned to combustible only (with 2.0% transitioning to cigarettes only, Table S4), 6.2% transitioned to combustible + noncombustible, and 12.8% transitioned to no tobacco use (Figure 1 and Table S2).

#### 3.2.4. Electronic Nicotine Delivery Systems Only Users at Wave 1 (Accounting for 1.1% of the Young Adult Population (Table S1) and 0.9% of the Older Adult Population (Table S2))

Among young adults, about 25% of ENDS only users at W1 were still ENDS only users at W2, 20.6% transitioned to combustible only, 21.0% transitioned to combustible + ENDS, and 30.7% transitioned to no tobacco use (Figure 1 and Table S1). Among older adults, more than 45% of ENDS only users at W1 were still ENDS only users at W2, 13.2% transitioned to combustible only (with 12.0% transitioning to cigarettes only, Table S4), 14.3% transitioned to combustible + ENDS, and 24.9% transitioned to no tobacco use (Figure 1 and Table S2).

#### 3.2.5. Combustible + Noncombustible Users at Wave 1 (Accounting for 2.6% of the Young Adult Population (Table S1) and 1.2% of the Older Adult Population (Table S2))

Among young adults, more than 30% of combustible + noncombustible users at W1 were still combustible + noncombustible users at W2, 16.8% transitioned to combustible only, 12.2% transitioned to noncombustible only, 9.4% transitioned to combustible + ENDS, 15.9% transitioned to combustible + noncombustible + ENDS, and 7.4% transitioned to no tobacco use (Figure 1 and Table S1). Among older adults, more than 35% of combustible + noncombustible users at W1 were still combustible + noncombustible users at W2, 26.2% transitioned to combustible only, 14.3% transitioned to noncombustible only, 5.2% transitioned to combustible + ENDS, 7.2% transitioned to combustible + noncombustible + ENDS, and 7.8% transitioned to no tobacco use (Figure 1 and Table S2).

#### 3.2.6. Combustible + Electronic Nicotine Delivery Systems Users at Wave 1 (Accounting for 6.2% of the Young Adult Population (Table S1) and 3.6% of the Older Adult Population (Table S2))

Among young adults, more than 40% of combustible + ENDS users at W1 were still combustible + ENDS users at W2, 34.9% transitioned to combustible only, 5.5% transitioned to ENDS only, 3.3% transitioned to combustible + noncombustible + ENDS, and 11.5% transitioned to no tobacco use (Figure 1 and Table S1). Among older adults, more than 40% of combustible + ENDS users at W1 were still combustible + ENDS users at W2, 41.9% transitioned to combustible only, 5.6% transitioned to ENDS only, 1.5% transitioned to combustible + noncombustible + ENDS, and 7.2% transitioned to no tobacco use (Figure 1 and Table S2).

#### 3.2.7. Combustible + Noncombustible + Electronic Nicotine Delivery Systems Users at Wave 1 (Accounting for 1.3% of the Young Adult Population (Table S1) and 0.3% of the Older Adult Population (Table S2))

Among young adults, more than 25% of combustible + noncombustible + ENDS users at W1 were still combustible + noncombustible + ENDS users at W2, 15.9% transitioned to combustible only, 25.3% transitioned to combustible + noncombustible, and 17.5% transitioned to combustible + ENDS (Figure 1 and Table S1). Among older adults, more than 25% of combustible + noncombustible + ENDS users at W1 were still combustible + noncombustible + ENDS users at W2, 20.1% transitioned to combustible only, 24.7% transitioned to combustible + noncombustible, and 20.8% transitioned to combustible + ENDS (Figure 1 and Table S2). For both young adults and older adults, the rates of transition from combustible + noncombustible + ENDS at W1 to no tobacco at W2 were suppressed due to the relative standard error of the estimates being greater than 30%. Further details on rates of transitions among the three broad tobacco product categories are shown in Table S1 for young adults and Table S2 for older adults.

### 3.3. Within-Person Transitions among User Groups Based on Six Tobacco Product Categories

**Figure 2:**
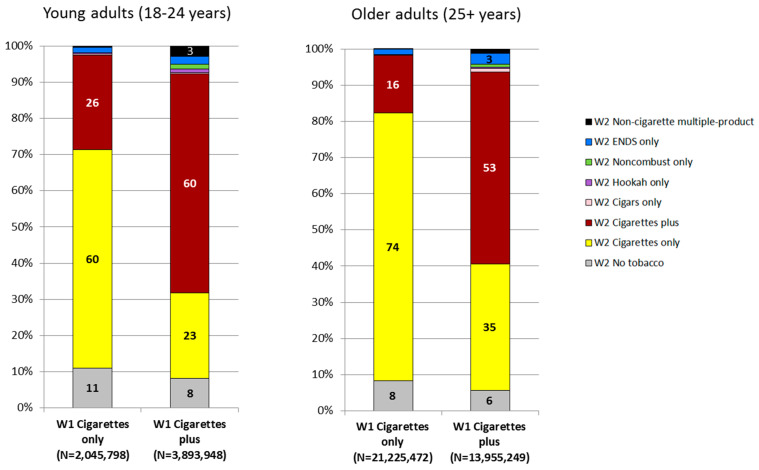
Transitions in current cigarette use among young adults (aged 18–24 years) and older adults (aged 25+ years) in the United States: 2013–2014 to 2014–2015. Notes. Each bar in the figure represents a cigarette user group at Wave 1 (W1). N underneath each bar indicates the weighted population size for each cigarette user group at W1 (i.e., the scale of the y-axis differs across bars). Transitions between W1 and Wave 2 (W2) are indicated by the colored distribution of each bar; for example, among the 2,045,798 young adult Cigarette only users at W1, 11% transitioned to No tobacco use at W2, 60% continued Cigarettes only use at W2, and 26% transitioned to Cigarettes plus use at W2. Current tobacco use defined as: for cigarettes, currently using everyday/somedays and smoked at least 100 cigarettes in lifetime; for hookah, currently using everyday/somedays/usually weekly/usually monthly; for all other products, currently using everyday/somedays. Single/multiple-product use defined with respect to the following six product types: cigarettes, cigars, hookah, pipe, noncombustible (i.e., smokeless tobacco, snus pouches, dissolvable tobacco), ENDS (which includes e-cigarettes at each wave and e-products at W2). ‘Cigarettes only’ defined as current use of cigarettes and no current use of any other product, ‘Cigarettes plus’ defined as current use of cigarettes plus current use of at least one other product, ‘Cigars only’ defined as current use of cigars and no current use of any other product, ‘Hookah only’ defined as current use of hookah and no current use of any other product, ‘Noncombustible only’ defined as current use of noncombustible and no current use of any other product, ‘ENDS only’ defined as current use of ENDS and no current use of any other product, ‘Non-cigarette multiple-product’ defined as current use of at least two product types excluding cigarettes, ‘No tobacco’ defined as no current use of any tobacco product. Estimates are suppressed when unweighted denominator <50 or RSE>30%. Non-suppressed estimates <3 are not shown in the figure (for readability) but are shown in the appendix.

Within-person transitions among user groups based on the six tobacco product categories as defined in Table 1 are presented for cigarette smokers in Figure 2, and for each of the other five tobacco product categories in Table S3 (young adults) and Table S4 (older adults).

#### 3.3.1. Cigarettes Only Users at Wave 1 (Accounting for 6.7% of the Young Adult Population (Table S3) and 10.4% of the Older Adult Population (Table S4))

Among young adults, 59.9% of cigarettes only users at W1 were still cigarettes only users at W2, 25.9% transitioned to cigarettes plus, and 11.0% transitioned to no tobacco (Figure 2 and Table S3). Among older adults, 73.7% of cigarettes only users at W1 were still cigarettes only users at W2, 15.9% transitioned to cigarettes plus, and 8.3% transitioned to no tobacco (Figure 2 and Table S4).

#### 3.3.2. Cigarettes Plus Users at Wave 1 (Accounting for 12.7% of the Young Adult Population (Table S3) and 6.8% of the Older Adult Population (Table S4))

Among young adults, 59.7% of cigarettes plus users at W1 were still cigarettes plus users at W2, 23.3% transitioned to cigarettes only, and 8.1% transitioned to no tobacco (Figure 2 and Table S3). Among older adults, 52.9% of cigarettes plus users at W1 were still cigarettes plus users at W2, 34.8% transitioned to cigarettes only, and 5.7% transitioned to no tobacco (Figure 2 and Table S4). Classification in the same tobacco user category in W2 as in W1 was generally much less common for non-cigarette tobacco products than for cigarettes (Tables S3 and S4, exception for noncombustible use among older adults, Table S4). For example, among young adult hookah only users at W1, only 22.4% were still hookah only users at W2 (Table S3), and among older adult hookah only users at W1, only 20.2% were still hookah only users at W2 (Table S4).

#### 3.3.3. Overall

Lastly, among those using any tobacco product or combination of tobacco products at W1, 50.6% (95% Confidence Interval (CI): 49.5–51.7) experienced any transition in product use or combination of use between W1 and W2 (data not shown in tables), with prevalence of any transition being higher for young adult tobacco users (71.7%, 95% CI: 70.0–73.4) than for older adult tobacco users (45.9%, 95% CI: 44.7–47.0).

## 4. Discussion

Findings from this study provide a broad look at the experience of adult tobacco users and nonusers in the U.S. in the context of the growing popularity of non-cigarette tobacco products, especially ENDS. Overall population prevalence of use of any tobacco product decreased over the one-year period, from 27.6% to 26.3%. This decrease in population-level tobacco use prevalence is a function of the magnitude of transitions among tobacco user groups within the context of the relative sizes of tobacco user groups at W1. Transitions in tobacco product use were common, especially in younger compared to older adults (72% vs. 46%), but for the most part, non-tobacco users at W1 remained non-tobacco users at W2, and tobacco users at W1 remained tobacco users at W2. Among young adults, cigarette use was most stable over the study period, and among older adults, cigarette use and noncombustible tobacco use were most stable. 

In recent years, the tobacco product landscape in the U.S. has expanded to include new products, not only within traditional product classes (e.g., cigars), but also within new product classes, such as the proliferation and evolution of ENDS [18,19,20]. Our findings show that, among young adult tobacco users at W1, 72% experienced any transition in use, including 21% who discontinued use completely. Among older adult tobacco users at W1, 46% experienced any transition in use, including 13% who discontinued use completely. These findings show that transitions in tobacco product use are age-related with more overall volatility seen in younger adults compared to older adults, indicating that for older adults, product use patterns tend to be more established and product experimentation tends to be less common than for young adults.

Our findings also show that there was greater stability in use of cigarettes compared to other tobacco product categories over the one-year study period. The prevalence of cigarette only users at W1 who were cigarette only users at W2 was generally higher (60% among young adults and 74% among older adults) than the prevalence of being classified in the same user group at both waves for any other single product user group (Tables S3 and S4), except for noncombustible only use among older adults (77%, Table S4), which was also relatively stable. In contrast, ENDS only use was relatively unstable; among young adult ENDS only users at W1, 42% transitioned to a tobacco product use group at W2 that included a combustible tobacco product, and 31% transitioned to no tobacco use at W2 (Figure 1).

The greater likelihood of continuing with/transitioning toward combustible use, particularly cigarette use, compared to continuing with/transitioning toward ENDS use is a key finding given the exceptional harmfulness of combustible tobacco use, with cigarettes killing half of all long-term smokers [1]. Our findings are consistent with long-known effectiveness of nicotine delivery for cigarettes and their associated abuse liability [21], and further, our findings suggest the persistence of cigarettes’ addictiveness [22] despite the changing tobacco product landscape in the U.S. and extensive transitioning in tobacco product use overall. As the U.S. Food and Drug Administration (FDA) moves forward with its regulatory framework based on a continuum of product risk [23], it is important to track how product use transitions shift over time, especially transitions between products with varying risk profiles. Reducing nicotine content in cigarettes, which could reduce addictiveness [24], along with increasing availability of potentially less harmful nicotine products, may facilitate transitions away from cigarette smoking among American adults.

While some national surveys (e.g., National Survey on Drug Use and Health, National Adult Tobacco Survey, National Health Interview Survey) document tobacco use incidence and prevalence in the U.S. and produce estimates that align with similarly defined estimates from the PATH Study [25,26,27], the longitudinal cohort design of the PATH Study allows us to track within-person changes in tobacco product use over time as the tobacco product and regulatory landscape continues to change. While we were limited to looking at aggregate tobacco product groups here (e.g., traditional cigars, cigarillos, and filtered cigars were grouped into a single cigar category) and were limited to looking at broad transitions in use, our findings can serve as a foundation for more in-depth analyses as the scientific community pursues a greater understanding of the potential impacts of emerging tobacco products [28,29,30]. To better understand population-level health consequences of tobacco use, each of the transitions that we report here can be further explored, keeping in mind the relative sizes of different tobacco user groups, and considered with respect to their implications for health. For example, additional research can examine the individual and co-occurring impacts of the amount and frequency of products used, flavors, nicotine content, past use history, dependence level, demographic characteristics, and reasons for product use.

## 5. Conclusions

W1 and W2 PATH Study data reveal extensive transitioning in non-cigarette tobacco product use, particularly among young adults, in contrast to the relative stability of cigarette smoking (and of noncombustible use among older adults), despite the changing tobacco product marketplace. Cigarette smoking still dominates tobacco use among adults in the U.S. Over 5 million young adults aged 18–24 years and over 38 million older adults aged 25+ years were current cigarette smokers in 2014–2015 (Tables S3 and S4), reinforcing the need for continued public health focus on cigarette smoking prevention and cessation efforts, including educating the public about the health risks of smoking.

## Figures and Tables

**Table 1:** Tobacco product user groups and cross-sectional weighted prevalence among adults at Wave 1 and Wave 2 of the Population Assessment of Tobacco and Health (PATH) Study.

User Group	Tobacco Product(s) Used	Young Adults (18–24 Years) W1 *N* = 9109 W2 *N* = 8174		Older Adults (25+ Years) W1 *N* = 23,194 W2 *N* = 20,183	
		W1% (SE)	W2% (SE)	W1% (SE)	W2% (SE)
Any current tobacco	Current use of any tobacco product	37.6% (0.885)	35.4% * (0.759)	26.0% (0.320)	25.0% * (0.312)
No current tobacco	No current use of any tobacco product	62.4% (0.885)	64.6% * (0.759)	74.0% (0.320)	75.0% * (0.312)
Never tobacco	Never use of any tobacco product	32.9% (1.078)	30.1% * (1.032)	25.6% (0.546)	25.1% * (0.563)
**Three broad tobacco product categories ^1^: Single-product use**					
Any single-product	Current use of only 1 of the 3 broad tobacco product categories	26.9% (0.691)	22.6% * (0.589)	20.2% (0.285)	19.7% * (0.304)
Combustible only	Current use of combustible and no current use of either of the other 2 broad product categories	24.4% (0.667)	18.5% * (0.518)	17.5% (0.265)	16.8% * (0.273)
Noncombustible only	Current use of noncombustible and no current use of either of the other 2 broad product categories	1.2% (0.120)	1.3% (0.132)	1.6% (0.073)	1.6% (0.093)
ENDS only	Current use of ENDS and no current use of either of the other 2 broad product categories	1.1% (0.108)	2.7% * (0.207)	0.9% (0.055)	1.3% * (0.079)
**Three broad tobacco product categories ^1^: Multiple-product use**					
Any multiple-product	Current use of any 2 or 3 of the 3 broad tobacco product categories	10.2% (0.368)	12.3% * (0.410)	5.1% (0.123)	5.1% (0.139)
Combustible + Noncombustible	Current use of combustible + current use of Noncombustible and no current use of ENDS	2.6% (0.174)	1.8% * (0.164)	1.2% (0.063)	0.8% * (0.050)
Combustible + ENDS	Current use of combustible + current use of ENDS and no current use of Noncombustible	6.2% (0.307)	9.1% * (0.377)	3.6% (0.104)	3.9% * (0.126)
Noncombustible + ENDS	Current use of Noncombustible + current use of ENDS and no current use of Combustible	#	0.2% (0.044)	#	0.0% (0.011)
Combustible + Noncombustible + ENDS	Current use of combustible + current use of Noncombustible + current use of ENDS	1.3% (0.104)	1.2% (0.088)	0.3% (0.031)	0.3% (0.034)
**Six tobacco product categories ^2^: Single-product use**					
Any single-product	Current use of only 1 of the 6 tobacco product categories	18.7% (0.497)	17.4% * (0.513)	16.7% (0.266)	17.5% * (0.297)
Cigarettes only	Current use of cigarettes and no current use of any of the other 5 product categories	6.7% (0.289)	7.0% (0.328)	10.4% (0.232)	12.2% * (0.241)
Cigars only	Current use of any cigar and no current use of any of the other 5 product categories	2.9% (0.179)	2.3% * (0.196)	2.3% (0.085)	1.9% * (0.111)
Hookah only	Current use of hookah and no current use of any of the other 5 product categories	6.4% (0.300)	3.7% * (0.311)	0.6% (0.051)	0.3% * (0.043)
Pipe tobacco only	Current use of pipe tobacco and no current use of any of the other 5 product categories	#	#	0.1% (0.020)	0.1% * (0.016)
ENDS only	Current use of ENDS and no current use of any of the other 5 product categories	1.1% (0.108)	2.7% * (0.207)	0.9% (0.055)	1.3% * (0.079)
Noncombustible only	Current use of any noncombustible tobacco and no current use of any of the other 5 product categories	1.2% (0.120)	1.3% (0.132)	1.6% (0.073)	1.6% (0.093)
**Six tobacco product categories ^2^: Dual-product use**					
Cigarettes + ENDS only	Current use of cigarettes and current use of ENDS and no current use of any of the other 4 tobacco product categories	1.4% (0.117)	2.3% * (0.194)	2.2% (0.073)	2.6% * (0.097)
**Six tobacco product categories ^2^: Multiple-product use**					
Any multiple-product	Current use of any 2 or more of the 6 tobacco product categories	18.3% (0.563)	17.2% * (0.467)	8.1% (0.135)	7.2% * (0.144)
Cigarettes plus	Current use of cigarettes + current use of one or more of the other 5 product categories	12.7% (0.472)	10.8% * (0.352)	6.8% (0.119)	6.3% * (0.145)
Cigars plus	Current use of cigars + current use of one or more of the other 5 product categories	11.1% (0.387)	7.4% * (0.280)	4.3% (0.107)	3.3% * (0.094)
Hookah plus	Current use of hookah + current use of one or more of the other 5 product categories	11.7% (0.494)	9.% * (0.399)	1.5% (0.073)	1.0% * (0.060)
Pipe tobacco plus	Current use of pipe tobacco + current use of one or more of the other 5 product categories	2.1% (0.166)	1.4% * (0.148)	0.8% (0.052)	0.6% * (0.042)
ENDS plus	Current use of ENDS + current use of one or more of the other 5 product categories	7.7% (0.325)	10.5% * (0.402)	4.0% (0.108)	4.2% (0.131)
Noncombustible plus	Current use of noncombustible + current use of one or more of the other 5 product categories	4.0% (0.203)	3.2% * (0.167)	1.5% (0.068)	1.2% * (0.052)

“W1” refers to Wave 1 of the PATH Study; “W2” refers to Wave 2 of the PATH Study. “SE” refers to standard error. *N* indicates the unweighted sample sizes at W1 and W2 for each age strata, which vary slightly across user groups due to missing data. W1 estimates were weighted using W1 cross-sectional weights and W2 estimates were weighted using W2 longitudinal weights; the W1 estimates represent the civilian, noninstitutionalized population at the time of W1; the population represented at W2 is those members of the U.S. civilian, noninstitutionalized population at W1 who are still residents of the U.S. in W2. ^1^ The three broad tobacco product categories are as follows: combustible (i.e., cigarettes, traditional cigars, cigarillos, filtered cigars, hookah, and pipe tobacco), noncombustible (i.e., smokeless tobacco, snus pouches, and dissolvable tobacco), and electronic nicotine delivery systems (ENDS). ^2^ The six tobacco product categories are as follows: cigarettes, cigars (i.e., traditional cigars, cigarillos, and filtered cigars), hookah, pipe tobacco, ENDS, and noncombustible tobacco (i.e., smokeless tobacco, snus pouches, and dissolvable tobacco). The ‘Never tobacco’ user group is a subset of the ‘No current tobacco’ user group. The dual-product user group overlaps with the multiple-product user groups. The multiple-product user groups necessarily overlap with one another. * indicates a statistically significant difference in prevalence between W1 and W2 (Chi-squared, *p* < 0.05). # estimate suppressed because it is statistically unreliable, based on a sample size of less than 50, or the coefficient of variation of the estimate is larger than 30%.

## Supplementary Materials

The following are available online at https://www.mdpi.com/1660-4601/15/11/2515/s1, Table S1: Transitions in current tobacco product use based on 3 broad tobacco product categories and considering single-/multiple-product use: Young adults 18–24 years of age, Table S2: Transitions in current tobacco product use based on 3 broad tobacco product categories and considering single-/multiple-product use: Older adults 25+ years of age, Table S3: Transitions in current tobacco product use based on 6 tobacco product categories and considering single-/multiple-product use: Young adults 18–24 years of age, Table S4: Transitions in current tobacco product use based on 6 tobacco product categories and considering single-/multiple-product use: Older adults 25+ years of age.
